# Urinoma Due to Spontaneous Rupture of the Renal Pelvis Mimicking Appendicitis

**DOI:** 10.7759/cureus.36141

**Published:** 2023-03-14

**Authors:** Rajat Mahawar, Jay D Dharamshi, Raju K Shinde, Chetna Rathi

**Affiliations:** 1 General Surgery, Jawaharlal Nehru Medical College, Datta Meghe Institute of Higher Education and Research, Wardha, IND; 2 Urosurgery, Jawaharlal Nehru Medical College, Datta Meghe Institute of Higher Education and Research, Wardha, IND

**Keywords:** srrp, appendicitis, extravasation, urinoma, renal pelvic rupture

## Abstract

Spontaneous rupture of the renal pelvis (SRRP) with urine extravasation is rare. This condition is primarily associated with an obstructing ureteric calculus. It creates a diagnostic dilemma, especially when the clinical diagnosis can be inconsistent. Herein, we report a 49-year-old male patient who presented with abdominal pain for the past three days and was diagnosed with acute appendicitis. A computed tomography (CT) scan revealed a right renal pelvis rupture and urinoma secondary to an obstructive 4 mm ureterovesical junction calculi. The patient was successfully treated with double-J stent placement. In conclusion, even though SRRP is rare, emergency physicians should have knowledge regarding this condition, which often presents as an abdominal condition and may be misdiagnosed as another condition requiring surgical intervention. Radiologic investigations such as CT scans are useful methods in suspected cases of this condition in order to reduce unnecessary surgical intervention.

## Introduction

Spontaneous rupture of the renal pelvis (SRRP) and subsequently urinoma is an underreported condition in the literature and represents less than 1% of renal injuries [1]. Extravasation of urine without recent ureteric instrumentation, recent surgery, external trauma, or renal tumors is known as SRRP [2]. Urine collections in the retroperitoneum, particularly in the perirenal region, which are typically caused by urinary tract leaks as a result of trauma, post-instrumentation, or blockage, are the key characteristics of urinomas. Urine leakage into the retroperitoneal area may cause perirenal fat to become inflamed locally [3].

The manifestation of urinoma as the first sign of obstructive uropathy is rare and can easily be misdiagnosed as another condition that requires surgical intervention [1,3,4]. In this case report, a patient presented to the emergency department (ED) with symptoms of appendicitis but was later diagnosed with urinoma caused by an obstructive distal ureteric stone.

## Case presentation

Patient information

A 49-year-old male came to the ED reporting right-sided lower abdominal pain that started three days ago and intensified two hours before his arrival. The pain originated at home, and there was no history of trauma. The patient experienced nausea but no signs of fever, chills, or urinary tract-associated symptoms. He had no significant past medical history and no prior or recent surgical procedures.

Clinical findings

On physical examination, he was oriented and cooperative and only had tenderness in the right iliac fossa. His vital signs were normal: blood pressure 120/80 mmHg; cardiac rate 90 beats per min; and body temperature 36.8°C. On abdominal examination, tenderness and rebound tenderness were present in the right iliac fossa. The patient was admitted for further evaluation and management with a provisional diagnosis of acute appendicitis on clinical examination.

Diagnostic assessment

Creatinine and blood urea nitrogen levels were found to be normal during a laboratory evaluation. Additionally, the C-reactive protein level was 0.4mg/dL, the liver function test was within normal limits, and the total blood count revealed a hemoglobin of 13.2 grams per deciliter, total leucocyte count of 8,900 mcL, and a platelet count of 199,000; all fell within normal limits. Urine analysis was found to be normal.

Plain abdominal X-ray was unremarkable, while ultrasound of the abdomen showed the right-sided dilated pelvicalyceal system, the appendix could not be visualized, and there was minimal fluid collection in the pelvis.

**Figure 1:**
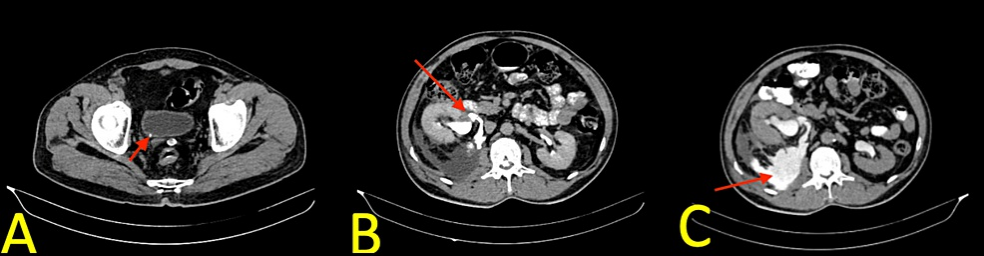
An abdominal computed tomography scan showing (A) a 4 mm calculus at the right vesicoureteral junction (arrow), (B) contrast leak from the renal pelvis (arrow), and (C) urinoma (arrow)

Utilizing IV contrast dye, a computed tomography (CT) intravenous urogram scan of the pelvis and abdomen was performed for further evaluation, and it revealed a 4 mm calculus at the right vesicoureteric junction (Figure 1A) causing proximal dilatation of ureter and pelvicalyceal system with extensive perinephric fat stranding and perinephric fluid collection. The fluid is tracking inferiorly along psoas muscle with evidence of contrast leak from the renal pelvis into the perinephric area in the delayed phase suggesting renal pelvis rupture (Figure 1B) and retroperitoneal urinoma (Figure 1C).

Diagnosis 

A provisional diagnosis of SRRP with urinoma was made depending on CT scan findings.

Therapeutic interventions

**Figure 2:**
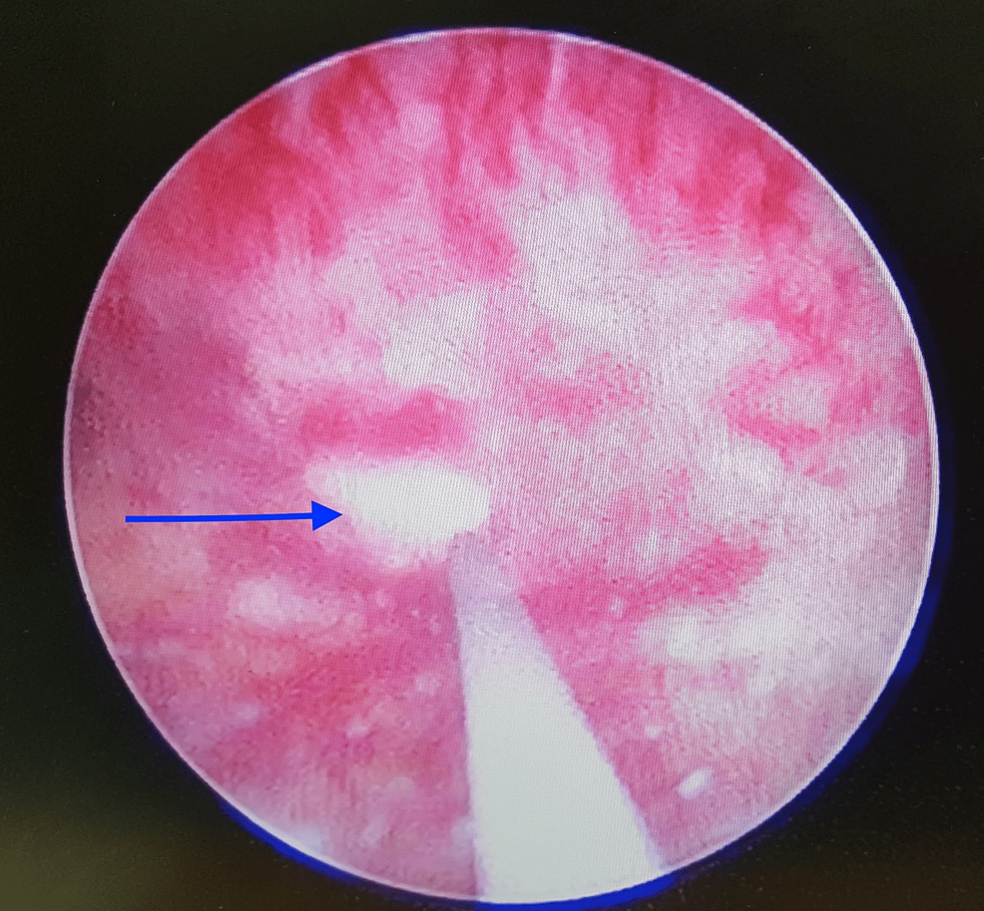
Site of pelvic rupture in the healing phase as seen on retrograde pyelography (arrow)

As a result, the patient was transferred to a urologist's care and given Inj Levofloxacin 500mg intravenously once a day, with adequate hydration as per the body weight. Due to the urinoma's small dimensions upon presentation and potential to reabsorb without surgery, the urologist decided to remove the obstruction and treat it conservatively. The patient underwent a double-J stent implantation after cystoscopy and retrograde pyelography. The pyelography showed a raw healing area of rupture (Figure 2). The procedure did not result in any immediate complications. 

Follow-up and outcome

The patient was discharged on day 2 and followed up in a urology clinic. The double-J stent was removed after 21 days without complication, and the patient follows up regularly in the outpatient clinic.

## Discussion

In the literature, SRRP is a disorder that is underreported. This is due to a number of factors such as contrast-enhanced imaging tests being not advised as the primary imaging techniques for the examination of patients with acute flank pain, and hence, clinically minor ruptures are likely to be left undiagnosed. The diagnosis of pelvic rupture and urinoma comes down in the list of differential diagnosis to be considered in case of left flank pain [5].

An unusual manifestation of ureteral stone obstruction is renal pelvis rupture; Wunderlich was the first to report it in 1856. The same etiologies that can induce pelvic rupture also contribute to forniceal rupture, which is a more common condition that is also related to obstruction [6].

Symptoms of pelvic rupture are similar to those of renal colic, e.g. nausea, flank pain, and vomiting. There is abdominal tenderness in physical examination, and there are signs of peritonitis; hence inflammatory conditions are to be primarily considered as differential diagnoses, e.g. appendicitis and cholecystitis [5,6].

SRRP is very unusual. The most often documented causes of rupture of the renal pelvis are stones, pregnancy, renal transplants, vesicoureteral reflux, iatrogenic trauma, ruptured renal cysts, tumors, strictures, retroperitoneal fibrosis, post-radiation scarring, retroperitoneal fibrosis, and urinary tract infections [7].

The literature underreports SRRP associated with distal ureteral stone. Ninety percent of 2-4 mm ureteral stones treated with observation therapy may pass on their own after 40 days. Fifty percent of stones larger than 5 mm may require treatment. In medical expulsive treatments in cases of distal ureteral stones with median sizes of 4.7-6.7 mm, 80% of the passage rate has been reported [8].

Urinoma, development of perinephric or retroperitoneal abscesses, and urosepsis are only a few of the catastrophic consequences that can result from spontaneous ureteral perforation [8,9]. So spontaneous ureter rupture needs to be attended to right away. Depending on the patient's condition and the grade of the ureteral rupture, treatment should be tailored [9].

Urinomas can be unilateral or involve both sides. Symptoms experienced by the patient can be due to encapsulated or confined collection. Primitively, researchers believed urinoma could possibly protect renal function. Recent studies, however, cast doubt on the protective effect of urinomas because some patients have reported a decreased renal function in the kidney on the same side (ipsilateral) as the urinoma. Urinomas can be obstructive or non-obstructive [10].

The causes of urinoma can be obstructive, such as pregnancy, pelvic masses, enlarged prostate, ureteral stones, enlarged lymphatic glands, post-radiation scarring, retroperitoneal fibrosis, congenital anomalies, and posterior urethral valves. Non-obstructive causes include injuries from genitourinary, retroperitoneal, pelvic, or gynecological surgeries, as well as kidney injuries. Urinoma is a rare disorder, often caused by trauma to the urinary tract in adults. Although urinary calculi and other obstructive conditions can lead to urinoma, they are less likely to be the direct cause. Pyelosinus backflow of urine, a condition that occurs when intrapelvic pressures exceed 35 cm H2O and the calyceal forniceal ruptures, can result in the development of urinoma. Urinary extravasation is present in 5% to 17% of cases of acute urinary blockage [7,10].

Urine frequently leaks with extensive extravasation into the perirenal or subcapsular region within Gerota's fascia, and it may also travel through lymphatic vessels. It will travel along the iliopsoas area just below the inguinal ligament to the buttocks, thighs, scrotum, or peritoneum if it extends inferiorly. The majority of the time, urinomas are modest when they are first discovered and will go away on their own. A urological procedure is frequently needed to remove a urinoma if there is a fever, a major damage, urosepsis, or a huge expanding urinoma that may be compressing. A CT KUB without contrast that includes a low-dose non-contrast phase and a delayed image phase performed 10 minutes after giving IV contrast for confirmation and analyzing a suspected perinephric collection may be used to examine the patient after renal ultrasonography. Management of spontaneous ureteral rupture is not standardized. Excellent results are achieved with conservative treatments and minimally invasive procedures [9,10].

Typically, the first-line treatment involves inserting a drain under imaging guidance into the urinoma and giving empiric antibiotics. A percutaneous nephrostomy tube may be inserted by the surgeon to improve drainage if the drain is unable to remove the urinoma as it should. A ureteral stent accelerates healing. In severe cases, surgery is performed. The best way to prevent extensive procedures is through early diagnosis and rapid treatment [11].

Only a tiny calculus, measuring 4 mm in diameter, was visible in our patient. Additionally, he had no prior trauma, so we had no reason to believe that his ureter would have ruptured. Although spontaneous ureteral rupture is a rare consequence of urolithiasis, when the calculi are tiny (<5 mm), it should still be taken into consideration as a differential diagnosis in emergency patients who have flank pain or pain in the right iliac fossa.

## Conclusions

Even though SRRP is rare, emergency physicians should have knowledge regarding this condition, which often presents as an abdominal condition and may be misdiagnosed as another condition requiring surgical intervention. Radiologic investigations such as CT scan are useful methods in suspected cases of this condition in order to reduce unnecessary surgical intervention. Conservative treatments, along with drainage techniques, may help to prevent further complications.
